# A Quality Control Check to Ensure Comparability of Stereophotogrammetric Data between Sessions and Systems

**DOI:** 10.3390/s21248223

**Published:** 2021-12-09

**Authors:** Kirsty Scott, Tecla Bonci, Lisa Alcock, Ellen Buckley, Clint Hansen, Eran Gazit, Lars Schwickert, Andrea Cereatti, Claudia Mazzà, on behalf of the Mobilise-D Consortium

**Affiliations:** 1 Department of Mechanical Engineering & INSIGNEO Institute of In Silico Medicine, The University of Sheffield, Sheffield S1 3JD, UK; t.bonci@sheffield.ac.uk (T.B.); e.e.buckley@sheffield.ac.uk (E.B.); c.mazza@sheffield.ac.uk (C.M.); 2 Translational and Clinical Research Institute, Faculty of Medical Science, Newcastle University, Newcastle upon Tyne NE4 5TG, UK; Lisa.Alcock@newcastle.ac.uk; 3 Department of Neurology, University Hospital Schleswig-Holstein, Campus Kiel, Kiel University, 24105 Kiel, Germany; c.hansen@neurologie.uni-kiel.de; 4 Centre for the Study of Movement, Cognition and Mobility, Tel Aviv Sourasky Medical Centre, Tel Aviv 6492416, Israel; erang@tlvmc.gov.il; 5 Department for Geriatric Rehabilitation, Robert-Bosch-Hospital, 70376 Stuttgart, Germany; lars.schwickert@rbk.de; 6 Department of Electronics and Telecommunications, Politecnico di Torino, 10129 Torino, Italy; acereatti@uniss.it

**Keywords:** optoelectronic stereophotogrammetry, 3D motion capture, quality control, spot check, accuracy, systematic errors, gait, human movement

## Abstract

Optoelectronic stereophotogrammetric (SP) systems are widely used in human movement research for clinical diagnostics, interventional applications, and as a reference system for validating alternative technologies. Regardless of the application, SP systems exhibit different random and systematic errors depending on camera specifications, system setup and laboratory environment, which hinders comparing SP data between sessions and across different systems. While many methods have been proposed to quantify and report the errors of SP systems, they are rarely utilized due to their complexity and need for additional equipment. In response, an easy-to-use quality control (QC) check has been designed that can be completed immediately prior to a data collection. This QC check requires minimal training for the operator and no additional equipment. In addition, a custom graphical user interface ensures automatic processing of the errors in an easy-to-read format for immediate interpretation. On initial deployment in a multicentric study, the check (i) proved to be feasible to perform in a short timeframe with minimal burden to the operator, and (ii) quantified the level of random and systematic errors between sessions and systems, ensuring comparability of data in a variety of protocol setups, including repeated measures, longitudinal studies and multicentric studies.

## 1. Introduction

Optoelectronic stereophotogrammetric (SP) systems are integral in the field of human movement research for quantifying the kinematic variables of human movement through the instantaneous 3D tracking of retroreflective or light emitting markers [1]. These systems have gained recognition in clinical application and diagnostics [2,3,4] and are regarded as the gold standard for the validation of other technologies with a similar purpose [5,6,7,8]. In fact, SP systems allow the tracking of marker trajectories with submillimetre accuracy and precision [9]. However, factors such as the number of cameras, camera resolution, camera positioning, laboratory environment and capture volume can affect the accuracy of these systems [10]. As a result, rigorous testing to attain a metrological characterisation and comprehensive understanding of the consistency in error between sessions and across systems is crucial for protocol designs such as, repeated measures, longitudinal studies and multicentric studies.

Although SP systems have an internal quality assessment of the random and systematic errors during the calibration procedures, the reporting varies between manufacturers and does not entail the provision of easily readable feedback. In response, standard procedures to metrologically characterise different SP systems, by quantifying the error in marker reconstruction, have been proposed. However, these methods can be complex and regularly require the use of bespoke equipment, with a cluster of markers attached to turning plates [11,12,13], sliding plates [14], rigid rods [15,16], sliding blocks allowing adjustable linear movement of the marker cluster [17,18,19], or articulated arms with 3 degrees of freedom [11] and regularly involve a time intensive assessment in respect to the number of recordings required (3–45 per check) and modifications in equipment setup between each trial [11,12,14,16,17,18,19]. Although the quantification of random errors is regularly reported in these methods, few have considered the quantification of systematic errors. Often this has been limited to a dynamic capture in the centre of the capture volume consisting of rotational movements [11,12,13,15,16], which may not represent the systematic errors accumulated in dynamic movements typically seen in human movement data.

In response, recent studies have considered the addition of dynamic checks more representative of human movement data. To this end, Eichelberger et al. [20] used a plate consisting of two markers attached to the foot, knee and sacrum during a straight walking trial to determine the error at the three most common heights of marker placement throughout the chosen volume of capture. Although this study clearly demonstrated the need for assessing systematic errors under dynamic conditions, the proposed setup requires a separate trial for each position of the plate and subsequently would involve additional time prior to each data collection, which may not always be available in clinical-based laboratories. Additionally, this approach did not consider errors in the estimates of angles between multiple markers, relevant when estimating angular kinematics. More recently, Di Marco et al. [21] proposed a quick dynamic check of systematic errors using the calibration object provided by the SP manufacturer as it moved through the capture volume for approximately 20 s. Their method led to similar results in a participant-based check (a maximum error of 0.7° for the object compared to 2.4° for the gait trials); however, validation of this method is restricted, since the reliability of the check across different systems, operators and calibration objects has not yet been assessed. Additionally, automation of the processing and reporting of errors was not implemented and its ability to ensure comparability as a routine quality control (QC) check in SP data collection across different laboratories has not been assessed.

Based on the encouraging results from Di Marco et al. [21], this study aims to develop and validate a simple and time effective QC check to estimate the random and systematic errors of different SP systems that can be adopted in the routine running of SP data collections, with minimal burden to the operator, no need for additional equipment and automated reporting of random and systematic errors. To determine the QC check’s reliability, robustness and ability to discriminate a change in systematic error between calibration/session, a validation will be completed in two parts to verify whether the QC check is (a) reliable, regardless of the calibration object used and (b) robust regardless of operator and system calibration/session. Additionally, to demonstrate its ability to ensure the comparability of SP data between systems, the proposed QC check will be deployed in a multicentric study that consists of five laboratories with varying SP systems and operators.

## 2. Materials and Methods

### 2.1. The Quality Control (QC) Check

**Figure 1:**
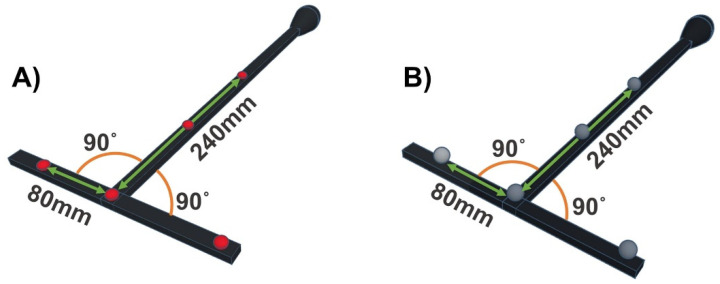
Schematic of the defined linear and angular marker geometries of *CO1*. (**A**) the active marker version of *CO1* (**B**) the passive marker version of *CO1*.

The proposed QC check makes use of the manufacturer’s calibration object (or a similar alternative, as described later in the methods) during two short acquisitions. The calibration object chosen for this study was the Vicon calibration wand (Vicon Motion Systems, Oxford, UK) (Figure 1), which will be referred to as *CO1* throughout. *CO1* was used for all data collection except the assessment of the QC check’s reliability with a different calibration object.

To first isolate random errors associated to the internal SP systems algorithms in marker reconstruction, the calibration object is positioned in the middle of the capture volume and a static trial of approximately 5 s is recorded. With this placement, the “ideal” capture can be quantified with minimum influence from calibration outcome, camera placement or intrinsic camera settings [14]. Subsequently, to quantify the systematic errors accumulated by the specific system setup and calibration outcome, the calibration object is moved through the capture volume at a velocity comparable with that used in the dynamic phase of the system calibration, for a minimum of 20 s or until the full capture volume has been covered.

### 2.2. Validation 

The reliability of the proposed QC check was assessed through a validation that included assessment of the three variables subject to change on its use: the calibration object used (Part a), the operator performing the trials and the system calibration/session (Part b). 

Data for assessing characteristics of the calibration object was collected using a 10 camera Vicon system T-Series (camera model: T160, camera resolution: 16 mpxs, capture volume: 6.0 m × 4.0 m × 2.1 m) with a sampling frequency of 100Hz and processed in Nexus 2.8.2 (Vicon Motion Systems, Oxford, UK). Prior to data collection, the system was calibrated with a minimum of 3000 frames successfully capturing the calibration object for each camera. All testing was completed at a single site, on the same day and with the same calibration.

As SP manufacturers’ calibration objects can vary between the use of retroreflective (passive) and light emitting (active) markers, the effects of using either marker type was assessed. To ensure consistency in marker configuration and object shape, two versions of *CO#1* were used to complete the QC check. A single run of the QC checks was completed with the active marker version of *CO#1* (Figure 1A) and then repeated using the passive marker version of *CO#1* (Figure 1B). 

Prior to quantification of the errors, the calibration objects marker configuration and known inter-marker distances and angles must be defined. For *CO#1*, these geometrical relationships include both linear and angular measures [21], considering both the shortest and longest marker distances (Figure 1). The known inter-marker geometries for this object were based on the measures given by the SP manufacturer.

**Figure 2:**
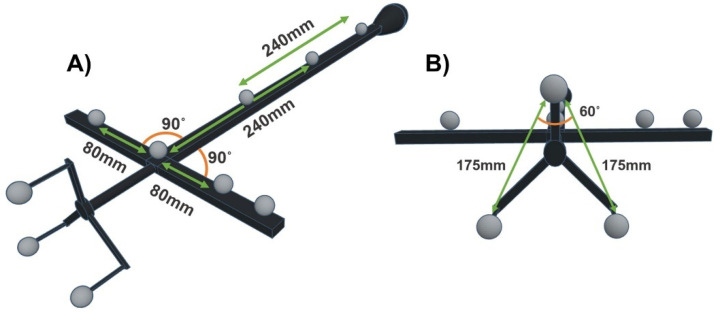
Schematic of the defined linear and angular marker geometries of *CO2* (**A**) defined marker geometries of the 2D marker configuration (**B**) defined marker geometries of the 3D marker configuration.

To determine the robustness of the QC check when using different calibration objects and marker configurations (Part b), the QC check was also performed using *CO2*. *CO2* was a modified version of the passive marker version of *CO1*, which consisted of two additional passive markers fitted to replicate the inter-marker distances in different locations of the object (Figure 2A). The additional markers were attached manually with the 3 repeated measures using a caliper to ensure accurate positioning. Furthermore, a 3D printed object that contained three passive markers was rigidly attached to the top of the object using stronghold tape, to determine the adaptability of the QC check to calibration objects with a third dimension in the marker configuration (Figure 2B). The dimension of the 3D object was specified in the manufacturing and as a sound check, the dimensions were then manually assessed using repeated measures of a caliper and goniometer. Using this approach should ensure the same level of accuracy as the dimensions of the original object from the manufacturer. 

To ensure the reliability of the QC check over repeated measures and determine if the QC check can identify a change in systematic errors between system calibrations/sessions as well as demonstrate the robustness of the QC check when performed by operators with varying levels of expertise, three conditions were considered: intra-operator intra-session, intra-operator inter-session, and inter-operator intra-session, respectively. All data were collected using the same SP system, specifications, and calibration procedure described above. The two intra-session conditions were completed with different system calibrations and on separate days. The active version of *CO1* was used for all data collections (Figure 1A).

The intra-operator intra-session condition was completed by the same operator (OP): *OP1*- highly experienced with the QC check procedures. Three repetitions of the QC check were performed during the same system calibration/session. The intra-operator inter-session was again completed by *OP1* with one recording completed on three different system calibrations/sessions. Finally, the inter-operator intra-session condition was completed by three different operators during the same system calibration/session. To evaluate the ease of performing the QC check and reliability of the outcome, the three operators had varying knowledge and experience with the check: *OP1, OP2—*had a good understanding of the QC check but did not perform it regularly, *OP3—*had no prior knowledge or use of the QC check. 

All operators were instructed to perform the trials using the same description and language as stated:

*Static Trial:* “Please place the calibration object level on the floor in the middle of the capture volume and record a trial of the object in this position for 5 s.”

*Dynamic Trial:* “Please move the calibration object at a velocity comparable with the one you would use in the system calibration procedure for at least 20 s. Please make sure to exploit the full volume of the desired capture area.”

### 2.3. Multicentric Deployment

**Figure 3:**
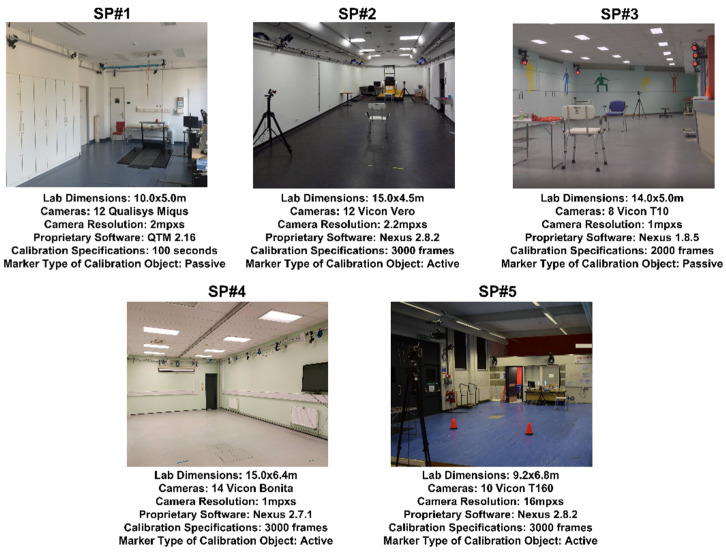
Specifications of each of the five SP systems (SP1–SP5) used in the multicentric deployment.

To evaluate its suitability in the context of a multicentric study, the QC check was implemented as part of an ongoing data collection for the IMI project Mobilise-D [22]. This study includes data collected from five different SP systems in different locations, with varying laboratory and system setups, SP system manufacturers and operators, as shown in Figure 3. The desired capture volume to be covered by all SP systems was defined as 5 m × 4 m × 2 m. Each site was instructed to calibrate its SP system following their standard procedures. Prior to implementation, all operators were trained by *OP1* on how to perform the QC check using the instructions described in the inter-operator intra-session protocol. All sites used the *CO1* object for this study (Figure 1), with either active or passive markers. Each laboratory completed the QC check on 10 different system calibrations all on different days of data collection.

### 2.4. Data Processing and Analysis

All data were reconstructed and labelled using the manufacturer software and recommendations. As adaptability to varying SP manufacturers was desired, a c3d file format was chosen for data export due to its universal use by different SP software. Using the calibration object’s marker trajectories, the distances and angles of the reconstructed markers were quantified. The error between the reconstructed and known inter-marker geometries defined above (Figure 1 and Figure 2) were calculated and the random and systematic errors were quantified as follows.

To characterise random errors accumulated from the SP marker reconstruction, the standard deviation of the error from the static trial was used to quantify the expanded uncertainty. By selecting a coverage of k=3, coverage of 99.7% of the random errors for a given session was obtained:

(1) $$\mathrm{Expanded}\;\mathrm{Uncertainty}=SD_{E}\times k.$$

The systematic error of the dynamic trial was calculated as the root mean square error (RMSE) of the difference between the known inter-marker geometries (y), as defined in Figure 1 and Figure 2, and the corresponding reconstructed inter-marker geometries ($\hat{y}$) for each frame of capture (i) over the full trial (N):

(2) $$\mathrm{RMSE}=\sqrt{\frac{\sum_{i=1\;}^{N}{\left(y_{i}-{\hat{y}}_{i}\right)}^{2}}{N}}$$

**Figure 4:**
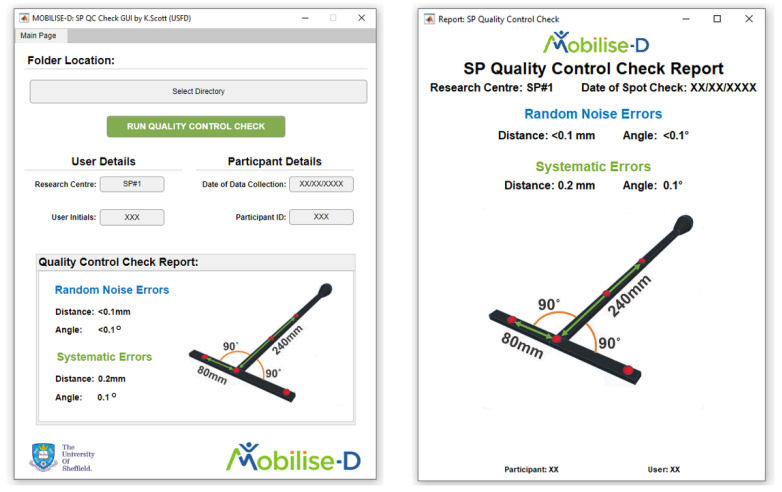
QC Check GUI created for multicentric deployment (**left**) and generated PDF reported (**right**).

To allow for immediate reporting of the QC check results, a graphical user interface (GUI) was designed in MATLAB 2020a (MathWorks, Natick, MA, USA) that reads the exported c3d files, compiles the calculation of the errors described above into an automatic pipeline and generates a report for straightforward interpretation of the errors by the operator (Figure 4). To allow amendment to any object and marker configuration, the base code used in the GUI for the QC check analysis and example data for both trials of the QC check is available as Data Availability Statement.

## 3. Results

Based on the initial analysis of the random and systematic errors, the quantified error did not show bias to differences in the distances or amplitude of the angle. Therefore, only the highest error for the inter-marker distances and angles are reported in the results.

### 3.1. Validation

In all testing completed for the variation in the calibration object (Part a), the random errors, as calculated by the expanded uncertainty, were below 0.1 mm for the inter-marker distances and below 0.1° for the angles. The systematic errors (RMSE) are reported in Table 1, with the highest RMSE for the marker distances of 0.8 mm and 0.5° for the angles.

In all testing for the variation in operator and session (Part b), the random errors (expanded uncertainty) showed the same results as seen in Part a, with the error of the inter-marker distances and angles always below 0.1 mm and 0.1°, respectively. The systematic errors, as quantified by the RMSE for the dynamic movement of the calibration object are reported in Table 2.

### 3.2. Multicentric Deployment

**Figure 5:**
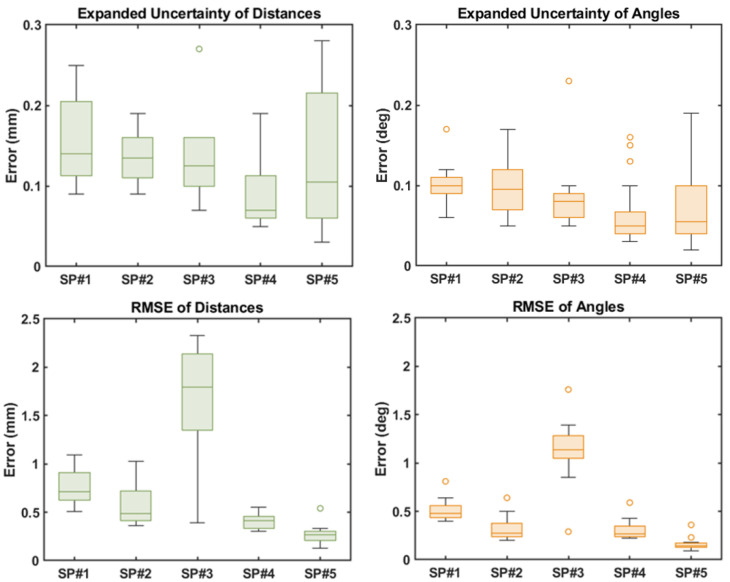
Box charts of the expanded uncertainty and RMSE calculated for the distances and angles of the 10 QC checks completed by the five sites.

The random errors (as calculated by the expanded uncertainty) for all 50 QC checks performed across the five SP systems, were always below 0.3 mm and 0.3° for the inter-marker distance and angles, respectively. The systematic errors (RMSE) quantified with the dynamic check are presented in Figure 5, with the highest RMSE for the inter-marker distances and angles below 2.5 mm and 2°, respectively.

## 4. Discussion

This study aimed to develop a simple and time effective QC check to estimate the random and systematic errors of different SP systems as part of the routine running of SP data collections. The reported results showed that by using the SP systems calibration object, with an assessment period of 25 s and automated calculation and generation of the errors, the proposed QC check can be successfully completed and interpreted well within 5 min. The validated QC check can be performed prior to starting an SP data collection and could be adopted with minimal delays or burden to the operator. The ability to perform such a check with no additional equipment is beneficial due to its wide implementation and routine use within standardised operating procedures to ensure accurate and reliable data collection.

### 4.1. Validation

The QC check was reliable in quantifying random and systematic errors between sessions regardless of the calibration object used, the operator performing the check and the system calibration/session.

The uncertainty quantification showed virtually no changes in the SP systems reconstruction of static inter-marker distances and angles regardless of object, marker type, operator or session. This demonstrates that using the middle of the capture volume as an “ideal” location for determining the random noise errors associated with reconstruction capabilities of the SP system is suitable and is minimally impacted by the calibration outcome. In addition, the errors quantified are comparable to previous studies that used bespoke equipment in determining random errors in a more structured manner [11,12,13,16,17,18,19].

The quantified systematic errors showed minimal change throughout the capture volume covered during the dynamic trials, with all errors at submillimetre and sub-degree level supporting the gold standard status of the system [9]. The range of systematic errors reported agree with previous methods that have used more structured assessments to consider the systematic errors at different static points of the capture volume [14] and are comparable with the errors quantified in the previously proposed dynamic checks reported by Di Marco et al. [21] (1.7 mm for inter-marker distances and 0.7° for angles) and Eichelberger et al. [20] (<1 mm error in inter-marker distances).

The slight increase in error observed for the calibration objects with passive markers could be explained by decreased precision of the reconstruction when compared to active markers [23]. Nonetheless, the quantified error fit the defined capabilities of an SP system and therefore can be considered negligible. The systematic errors quantified for the three operators with varying experience showed negligible differences (0.1 mm for distances and 0.1° for angles). Additionally, the QC check accurately identified the systematic errors related to changes in the system calibration while remaining precise across repeated measures, as shown in the results for the intra and inter-sessions.

### 4.2. Multicentric Deployment 

The deployment of the QC check as part of a multicentric study that included a variety of systems proved to be successful, with all sites smoothly completing the checks and interpreting the results prior to data collection in a time efficient manner (i.e., within the five-minute window stated above). In addition, the use of the accompanying GUI provided an automatic pdf export of the QC check report that could be appended with the SP data collected, to ensure easy reference and transparency of the systematic errors across sites and sessions. 

As shown in the quantification of the random errors for the 50 static trials acquired, the expanded uncertainty for the inter-marker distances and angles were all below 0.3 mm and 0.3° respectively (Figure 5), corroborating the findings from Di Marco et al. [21]. Although the level of random errors observed in the multicentric deployment was found to be slightly higher than in the validation (maximum difference of <0.2 mm for the marker distances and <0.2° for the angles), the error showed negligible change both within and between SP systems. Moreover, as the calibration object used (*CO1*) was the same in shape and marker configuration across all five sites (Figure 1), the slightly higher quantification of errors is likely due to the variation in camera specifications (e.g., number of cameras, camera resolution and camera placement) as well as the internal algorithms for marker reconstruction varying between the SP manufacturers. This supports the concept of the QC check being able to quantify and isolate random errors specific to a variety of systems.

The precision of the systematic errors quantified across the 10 sessions of data collection for each site showed minimal levels of change, with all reported errors comparable to the dynamic errors reported in previous studies [20,21], with the exception of *SP3* (Figure 5). As the random errors quantified for *SP3* in the middle of the capture volume fell into a similar range as the other SP systems, a possible reason for the higher systematic error and variation of this error between sessions is the limitation of having only eight cameras covering the defined capture volume of 5 m × 4 m × 2 m when compared to the other systems that ranged from 10–14 cameras, as well as a smaller camera resolution of 1 mpxs. Operation of an SP system with a smaller number of cameras has previously been shown to increase the level of systematic error and decrease the precision of marker tracking [14]. Moreover, as the lab size was comparable to two of the other systems used in this study (*SP2* and *SP4*), *SP3’*s higher systematic errors are considered to be primarily due to the limited ability of the fewer cameras to cover the full capture volume. Nonetheless, for the broader aim of the multicentric study, for which the experiments included in this paper were run [22], as spatiotemporal gait parameters are the primary focus from the gait data collected, a maximum linear error of 2.4 mm and angular error of 1.8° is certainly in an acceptable range when scaled to the quantified outputs (e.g., stride length, walking speed and turning angle).

The main limitation of the proposed QC check is the dependency of accuracy in the calibration object manufacturing and the assumption that there has been no deformation of object during standard use. Any inaccuracy of this sort, however, would also affect the system calibration and performance. In addition, due to the SP systems available in this study, only two SP manufacturers have been tested. However, with the source code of the GUI made available and the use of the universal c3d file, it is hoped that other systems could also use this check to produce a wider understanding of errors across different manufacturers.

## 5. Conclusions

This study clearly proved that the proposed QC check is feasible to perform in a short timeframe with minimal burden to the operator. It has a clear potential to be used as a routine procedure in multisession and multicentric studies. Its wide adoption will hopefully be boosted using the provided code, available in the Data Availability Statement.

## Figures and Tables

**Table 1:** The systematic error (RMSE) calculated for the single trial for each of the different calibration objects.

	RMSE			
	*CO1*		*CO2*	
Measure	Active Markers	Passive Markers	2D Configuration	3D Configuration
Distance (mm)	0.2	0.5	0.6	0.8
Angle (deg)	0.1	0.3	0.5	0.4

**Table 2:** Mean and standard deviation of the systematic error (RMSE) for the three trials for each of the different operator and session conditions.

	RMSE		
Measure	Intra-Operator Intra-Session	Intra-Operator Inter-Session	Inter-Operator Intra-Session
Distance (mm)	0.2 ± 0.1	0.3 ± 0.1	1.0 ± 0.1
Angle (deg)	0.1 ± 0.1	0.1 ± 0.1	0.4 ± 0.1

## Data Availability

The source code for the GUI developed for this study (which includes data extraction, processing, and automatic reporting of the QC check) as well as example data are available online at https://doi.org/10.15131/shef.data.16780678 accessed on 8 December 2021. The source code and example data are agnostic to SP system and manufacturer.
